# Double Competitive Immunodetection of Small Analyte: Realization for Highly Sensitive Lateral Flow Immunoassay of Chloramphenicol

**DOI:** 10.3390/bios12050343

**Published:** 2022-05-17

**Authors:** Dmitriy V. Sotnikov, Lyubov V. Barshevskaya, Anastasia V. Bartosh, Anatoly V. Zherdev, Boris B. Dzantiev

**Affiliations:** A.N. Bach Institute of Biochemistry, Research Center of Biotechnology of the Russian Academy of Sciences, Leninsky prospect 33, Moscow 119071, Russia; lyubov.barshevskaya@yandex.ru (L.V.B.); bartoshlab@yandex.ru (A.V.B.); zherdev@inbi.ras.ru (A.V.Z.)

**Keywords:** chloramphenicol, lateral flow assay, honey, mathematical modeling

## Abstract

A new scheme of reagents interaction for lateral flow immunoassay (LFIA) is proposed, which combines the features of competitive and sandwich assay and provides highly sensitive detection of low-molecular-weight analytes. Namely, the antigen in the sample interferes with the formation of the antibody (on the membrane)–hapten-protein–antibody (on the nanoparticle-marker) complex, competing with hapten-protein conjugate in both reactions. The proposed scheme was modelled using COPASI software, with a prediction of limit of detection (LOD) decrease by one order of magnitude compared to the standard competitive LFIA. This feature was experimentally confirmed for the detection of chloramphenicol (CAP) in honey. When tested in spiked honey, the visual LOD was 50 ng/mL for the common scheme and 5 ng/mL for the proposed scheme. Instrumental LOD was 300 pg/mL (1.2 µg/kg in conversion per sample weight of honey) in the standard scheme and 20 pg/mL (80 ng/kg in conversion per sample weight of honey) in the proposed scheme.

## 1. Introduction

Rapid detection of different analytes is a task in laboratory practice [1,2,3]. Simple, user-friendly, and low-cost point-of-care tests provide timely results and data processing. Lateral flow immunoassay (LFIA) is a prospective method that meets the aforementioned requirements: its duration is no more than 15 min, the assay is implemented without additional equipment, and it can be performed by non-qualified personnel [4,5,6]. Recent reviews demonstrate the wide use of LFIA to detect high-molecular-weight analytes (proteins, nuclear acids, etc.), corpuscular analytes (bacteria, viruses) [7], and low-molecular-weight analytes (toxins, antibiotics, drugs, etc.) [8,9,10]. However, a significant limitation of LFIA is the high limit of detection (higher than for equilibrium immunoassays such as ELISA) [11], which could be a critical parameter for an adequate conclusion regarding the presence or absence of an analyte in a sample [12,13]. At the same time, the requirements for sensitivity of analytical systems are constantly increasing. This is attributed to both new regulations (for example, in the field of safety) and the necessity of eliminating matrix effects of samples such as food and body tissues. For this reason, the requirements for assay sensitivity often exceed the standard regulations. This ensures a high demand to enhance the analytical sensitivity of LFIAs.

To determine antigen-antibody complex formation in small molecules, analytical systems implement competitive schemes, but these have their own limitations. One of the main problems related to competitive LFIA is insufficient assay sensitivity. In order to provide a lower limit of detection (LOD), it is necessary to reduce the concentration of specific antibodies. However, this decreases the amount of bound marker, and as a result, affects the intensity of analytical signal, which makes it difficult to detect the results. Another factor affecting assay sensitivity is limitations in antibody affinity [14]. The use of new nanoparticles or additional stages of signal amplification can improve sensitivity. However, these actions typically make assay implementation or registration of its results more complicated, which is not suitable for point-of-care testing [15,16,17,18]. As an alternative, several non-competitive techniques for small-molecule immunoassays have been proposed [19,20,21], but these assays need special reactants, such as antibodies, to be specific solely to the immune complex but not to its compounds. Because of this, the given techniques have been successfully realized for only some analytes.

In this study, we present a new scheme of competitive LFIA with higher sensitivity, which combines the features of common competitive and sandwich LFIA. The proposed scheme uses specific antibodies, immobilized on the surface of gold nanoparticles (GNP) and on the analytical zone of a nitrocellulose (working) membrane. Colored immune complexes in the analytical zone are formed due to polyvalent interactions of hapten-protein. If the target analyte is present in the sample, it blocks the binding sites of antibodies on the surface of GNP and on the analytical zone. As a result, two competitive reactions cause the change of detected signal. In our study, the given reactions were initially considered by mathematical modeling using COPASI software, and the decrease of LOD compared to the common competitive scheme was predicted. The stated feature of the proposed LFIA scheme was then confirmed experimentally.

Chloramphenicol (CAP), a broad-spectrum antibiotic, was chosen as the target analyte for these experiments. Nowadays, the use of CAP is prohibited due to its bone marrow toxicity, hepatotoxicity, associated reproductive disorders, and so on [22,23]. Despite these limitations, the use of CAP as a low-cost and effective medication in beekeeping is still being continued [24]; hence, the control of residual amounts of CAP in honey is highly in demand. In this regard, the development of a highly sensitive, rapid, and simple assay for CAP is an urgent task.

## 2. Materials and Methods

### 2.1. Reagents and Materials

Tetrachloroauric (III) acid, Tween-20, chloramphenicol succinate sodium salt (CAP-Su), chloramphenicol (https://www.sigmaaldrich.com/RU/ru/substance/chloramphenicolsuccinatesodiumsalt44518982570, accessed on 16 May 2022) (Sigma-Aldrich, St. Louis, MO, USA), sodium citrate (Reachem, Moscow, Russia), bovine serum albumin (BSA) (Boval Biosolutions, Cleburne, TX, USA), N-(3-dimethylaminopropyl)-N′-ethylcarbodiimide hydrochloride (EDC), sulfo-N-hydroxysuccinimide (sulfo-NHS) (Fluka, Buchs, Swizerland), 1-cyclohexyl-3-(2-morpholino-ethyl)carbodiimide metho-p-toluene sulphonate (CCMS), N,N-dimethylformamide (DMF) (MP Biomedicals, Santa Ana, CA, USA), and goat anti-mouse antibodies (Arista Biologicals, Allentown, PA, USA) were used in the study. Mouse monoclonal antibodies to chloramphenicol (clone B10) were kindly provided by Professor P. G. Sveshnikov (All-Russian Research Center of Molecular Diagnostics and Therapy, Moscow, Russia). The used salts, acids, and alkali were of analytical or chemical purity.

For the preparation of GNP solutions and their conjugates, deionized water (18 MΩ·cm at 25 °C) was prepared using the Simplicity system (18 MΩ·cm at 25 °C; Simplicity, Millipore, Burlington, MA, USA). 

Mdi Easypack (Advanced Microdevices, Ambala Cantt, India) set of membranes, including plastic backing L-P25; nitrocellulose (working) membrane CNPH90 with a pore size of 15 µm; conjugate application membrane PT-R5; sample application membrane FR1(0.6); and absorbent membrane AP045 were used for assembling test-strips for LFIA.

### 2.2. Equipment

IsoFlow automatic dispenser (Imagene Technology, Lebanon, NH, USA) was used to apply the reagents on the membranes. After assembling the membrane components, they were cut into strips 3.5 mm wide with an automatic guillotine cutter Index Cutter-1 (A-Point Technologies, Gibbstown, NJ, USA) and packaged in laminated aluminum foil using a mini-conveyor FR-900 (Wenzhou Dingli Packing Machinery, Wenzhou, China) with 0.5 g of packaged silica gel as a drying agent. Cutting and packaging were carried out at 20–22 °C in a special room with relative humidity of no more than 30%.

### 2.3. Synthesis of Hapten-Protein Conjugates

Conjugates of a modified derivative of chloramphenicol, CAP-Su with BSA, were obtained through carbodiimide-succinimide method according to [25]. The molar ratio of the obtained CAP:BSA conjugate was 20:1. Concentration of the conjugate was determined by measuring its optical density at 280 nm and comparing with the initial BSA solution using the UV-1202 spectrophotometer (Shimadzu, Kyoto, Japan). The dialyzed conjugate was stored at −20 °C.

### 2.4. Synthesis of Gold Nanoparticles (GNP) and Their Conjugates with Monoclonal Antibodies to CAP

The GNPs were synthesized according to Frens [26]. First, 1 mL of 1% HAuCl_4_ aqueous solution was added to 100 mL of water, after which the resulting mixture was brought to boil. Then, 1.5 mL of 1% aqueous sodium citrate solution was added with continuous stirring. The solution was kept boiling for 20 min, then cooled and stored at 4 °C.

The GNP conjugates with antibodies to CAP were synthesized according to [27]. Briefly, the antibodies were added to the GNP solution (OD_520_ = 1.0, pH 8.5) at concentrations of 10, 5, 2.5, 1.25, and 0.6 μg/mL and incubated with constant stirring for 30 min. BSA was added to the obtained solution to a final concentration of 0.25% and incubated for 10 min. The resulting conjugate was centrifuged for 15 min at 13,400 g. The precipitate was resuspended in 50 mM phosphate buffer (pH 7.5) containing 0.25% BSA. The obtained conjugate was stored at 4 °C.

### 2.5. Assembling of Test Strips for LFIA

GNP conjugate with anti-CAP antibodies was absorbed on a conjugate application membrane from a solution with OD_520_ = 2.0 in the amount of 16 μL per cm of the strip.

In a common competitive LFIA scheme, analytical zone was formed with CAP-BSA conjugate (20:1 mol/mol). Then, 2 μL of the conjugate (1.0 mg/mL in 50 mM PBS, pH 7.4) was applied per 1 cm of the strip.

In the proposed scheme, antibodies at a concentration of 1 mg/mL were immobilized on the analytical zone. Then, 2 μL of the conjugate (1.0 mg/mL in 50 mM PBS, pH 7.4) was applied per 1 cm of the strip. CAP-BSA conjugate was applied on the sample application membrane with a concentration of 0.2 μg/mL.

The control zone in both schemes was formed with goat anti-mouse antibodies at a concentration of 1 mg/mL. Then, 2 μL of the conjugate (1.0 mg/mL in 50 mM PBS, pH 7.4) was applied per 1 cm of the strip.

The membranes with the applied reagents were dried at 20–22 °C for 24 h.

### 2.6. Lateral Flow Assay of CAP

LFIA was carried out at room temperature. A standard solution of CAP was added to 50 mM PBS (pH 7.4) containing 0.25 % Tween-20 (PBST) and to samples of floral honey (the Novgorod Region, Russia) diluted with PBST in a ratio of 1:4, to achieve its final concentration in the range of 0.005–500 ng/mL. Test strips were placed into the samples in a vertical position, incubated for 15 min, and then removed and placed on a horizontal surface. The LFIA results were evaluated visually. The obtained test strips were scanned, and the color intensity in the analytical zones was determined using TotalLab TL120 (Nonlinear Dynamics, Newcastle, UK). The limit of detection was determined based on the obtained sigmoid dependence as the concentration corresponding to the signal and differing by three standard deviations from the background signal, according to existing recommendations [28]. The dependences of color intensity from the chloramphenicol concentration in the sample were plotted using Origin 9.0 software and approximated using the four-parameter sigmoid function:

$$y=A2+\left(A1-A2\right)/\left(1+{\left(x/x_{0}\right)}^{p}\right)$$

## 3. Results

### 3.1. Mathematical Description of Competitive LFIA Schemes

Competitive LFIA can be implemented in two basic formats: with labeled antibodies and with labeled antigens [29]. The most commonly used scheme with labeled antibodies is described below.

**Figure 1:**
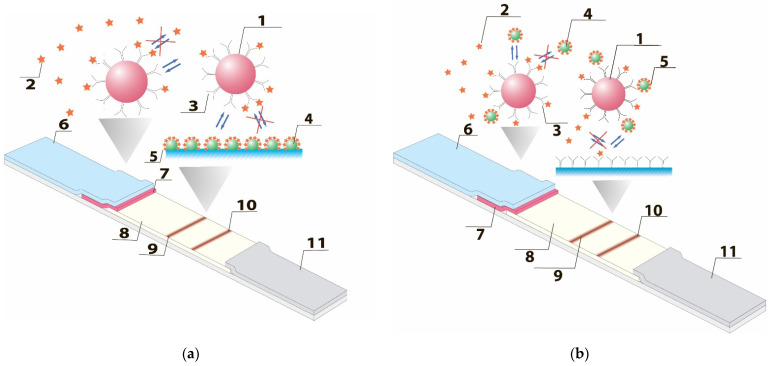
Scheme of the (**a**) standard and (**b**) proposed alternative competitive LFIAs. 1—Gold nanoparticles; 2—antigen in the sample; 3—antibodies against the antigen; 4—carrier protein; 5—antigen conjugated with carrier protein; 6—sample application membrane; 7—conjugate application membrane; 8—working nitrocellulose membrane; 9—analytical zone; 10—control zone; 11—final adsorbing membrane.

Labeled antibodies (P) and hapten-protein conjugate (R) are immobilized on the membrane composite. If the tested sample does not contain the target analyte (A), labeled antibodies bind with the hapten-protein conjugate, forming a complex (PR), which is visualized as a colored line. If the sample contains the target analyte, it binds with the labeled antibodies, forming the AP complex, and prevents the interaction of labeled antibodies with the immobilized hapten-protein conjugate on the analytical zone. As a result, no colored lines are formed on the analytical zone (see Figure 1a).

According to a standard competitive LFIA, two reactions take place: (1) A + P = AP (which occurs when a sample containing an analyte comes into contact with labeled antibodies) and (2) P + R = PR (which starts after the liquid sample reaches the analytical zone). The signal is proportional to the AP complex concentration [30]. The kinetics of these independent reactions are described by the differential equation system presented in the Supplementary Materials. A complete analytical and numerical mathematical description of competitive LFIA is provided in our previous work [30].

In this work, we propose the alternative scheme of competitive LFIA in which specific antibodies to target the analyte are immobilized both on the surface of GNP (P) and in the analytical zone (R). The hapten-protein conjugate is applied on the sample membrane (C). When the liquid sample is placed on the test strip, first, it combines with component C, and then with component P. As a result, the PC complex is formed. Components of the liquid sample move along the test strip through capillary force, and in the analytical zone, immobilized antibodies (R) bind to the PC complex due to several valencies of the PC. As a result of this interaction, a PCR complex is formed in the analytical zone. Alternatively, the formation of this complex can also occur as follows: initially, R interacts with unreacted C, then obtained CR complex reacts with P. If the target compound is present in the sample (A), it reacts with P, blocking the possibility of P binding to C. As the liquid sample reaches the analytical zone, A blocks R, additionally reducing the possibility of the formation of PCR complex (see Figure 1b). The overall scheme of this system consists of seven reactions: (1) A + P = AP; (2) P + C = PC; (3) P + PC = PCP; (4) A + R = AR; (5) C + R = CR; (6) PC + R = PCR; (7) P + CR = PCR. The signal in the proposed scheme is determined by the concentration of PCR complex. Consequently, compared to the common competitive LFIA, in the proposed LFIA, the analyte blocks not one, but two binding reagents. As a result, its influence on the quantity of the detected complexes increases, whereas LOD decreases. The labeled antibodies can bind to multivalent hapten-protein conjugate with the formation of PCP complex.

The abovementioned PCR complex formation in the proposed LFIA was simulated by applying numerical methods using the COPASI software package. The system of equations describing the change in the concentrations of reagents is presented in the Supplementary Materials. To describe the interactions in the systems, the following parameters were used: the association (k_ai_) and dissociation constants (k_di_) of each reaction; the total time of analysis, starting from the contact of the sample with labeled antibodies (t); and the time of sample migration from the applied labeled antibodies to the analytical zone (T) [30]. To compare the two systems, identical parameters were used.

**Figure 2:**
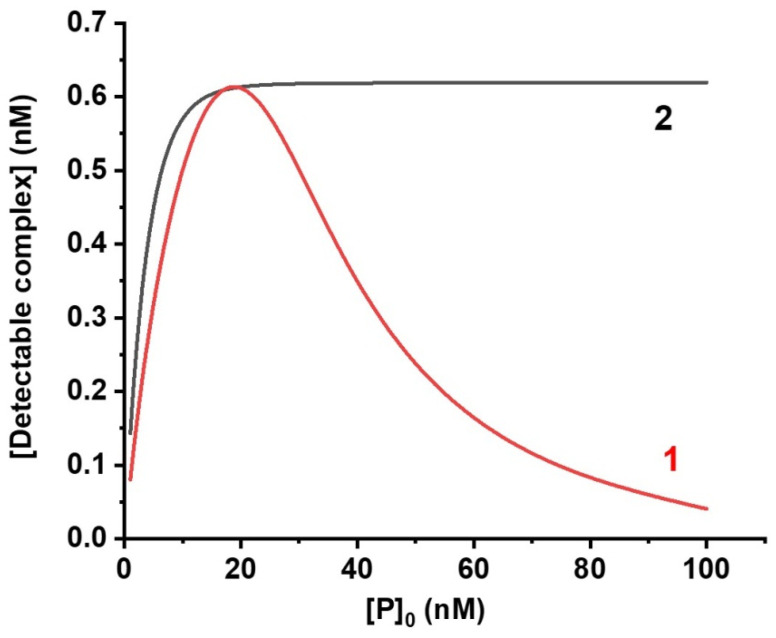
Dependence of the concentration of the detected complex in the absence of the analyte in (1) the proposed double competitive LFIA and (2) standard competitive LFIA. Model parameters: T = 30 s; t = 300 s; k_ai_ = 10^6^ 1/(M × s); k_di_ = 10^−4^ 1/s.; [A]_0_ = 0 M. 1—[C]_0_ = 10 nM; [R]_0_ = 1 nM; 2—[R]_0_ = 6.2 nM.

Theoretical and experimental studies of standard competitive LFIA show that reducing the concentration of labeled antibodies increases assay sensitivity and decreases color intensity of the analytical zone [30]. Consideration of the proposed double competitive LFIA demonstrated that dependency of the detected signal (quantity of PCR complex) on [P]_0_ (0 index means initial concentration) has a maximum, that is, an optimum concentration of labeled antibody at which the maximum concentration of the detected complex is reached (Figure 2). When using minimum specific antibody concentration conjugated with GNP, the effective binding of the analyte in the sample would not be reached. Therefore, during the formation of PCR complex in the analytical zone, the signal intensity will decrease. The use of maximum specific antibody concentration conjugated with GNP will block the formation of PCR complex in the analytical zone due to the formation of PCP complex during the binding of analyte with two conjugates of GNP with specific antibodies instead. In the double competitive LFIA scheme, the signal also depends on hapten-protein (C) concentration. Theoretically, the concentration [C]_0_ close to the optimum is approximately half of [P]_0_. Thus, Figure 2 shows that at an initial concentration of [C]_0_ equal to 10 nM, the maximum concentration of the detected complex is reached at a [P]_0_ of about 20 nM.

**Figure 3:**
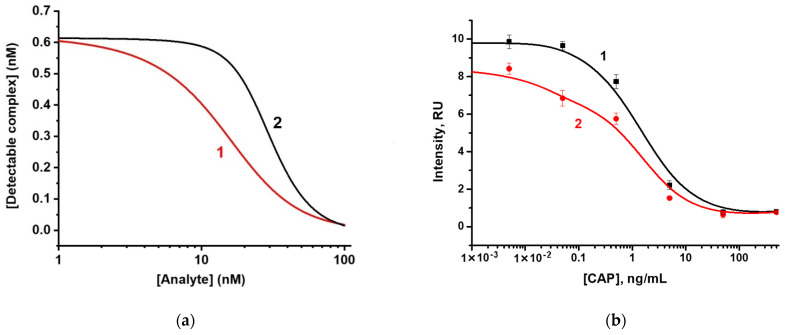
(**a**) Theoretical calibration curves for (1) double competitive LFIA and (2) standard competitive LFIA. Model parameters: T = 30 s; t = 300 s; k_ai_ = 10^6^ 1/(M × s); k_di_ = 10^−4^ 1/s. 1—[C]_0_ = 10 nM; [P]_0_ = 20 nM; [R]_0_ = 1 nM; 2—[P]_0_ = 20 nM; [R]_0_ = 0.62 nM. (**b**) Color intensity in the analytical zones of test strips (from Figure 5) for the determination of CAP in the (1) standard scheme of competitive LFIA and (2) proposed scheme of double competitive LFIA (**b**).

In standard competitive LFIA, the concentration of the receptor in the analytical zone [R]_0_ mainly affects the signal intensity, but it does not shift the calibration curve as far, as the formation of complex linearly depends on [R]_0_ [30]. In contrast, the proposed scheme [R]_0_ affects both signal intensity and assay sensitivity. To compare the LODs of two schemes, the chosen (varied) assay conditions should provide approximately equal label binding in the absence of an analyte, whereas the parameters of immunoreagents should be the same. Taking into account the identical [P]_0_ and selected [R]_0_ for such a unification of color intensity for both schemes, the numerical modeling demonstrated—see Figure 3—that the proposed double competitive LFIA provides better assay sensitivity due to the broadening of a working range of the calibration curve towards lower concentrations.

### 3.2. Optimization of LFIA Parameters to Perform Assay of CAP

To confirm the theoretical predictions of decreasing assay LOD in LFIA, the test systems for detection of CAP in standard and double competitive format were prepared and experimentally characterized.

**Figure 4:**
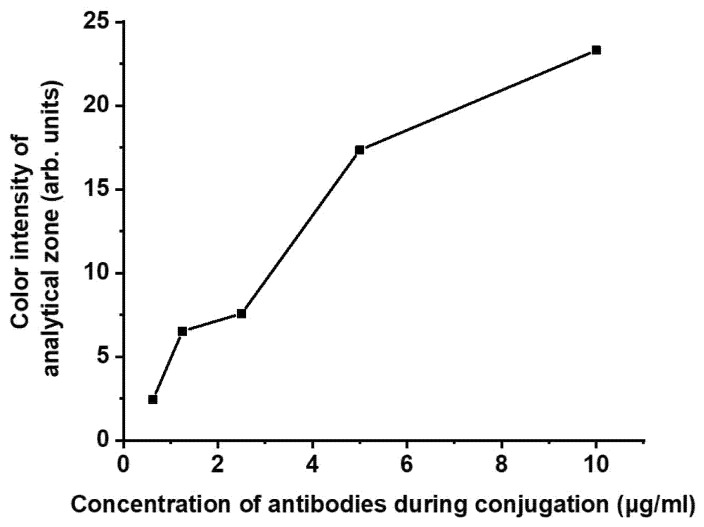
Dependence of the color intensity of the analytical zones on the concentration of antibodies when conjugated with a label in the standard competitive LFIA scheme in the absence of the analyte.

Since labeled antibody concentration was stated as the parameter influencing the assay sensitivity, we have studied the dependence of color intensity in the analytical zone on the concentration of antibodies conjugated with nanoparticles. The obtained results (Figure 4) demonstrate that decreasing antibody concentration leads to a decrease in maximal coloration in the analytical zone, but the assay sensitivity is increased [25,27]. Thus, to compare the LOD of both schemes, we have implemented the analyses under conditions providing a similar color intensity of the analytical zone in the absence of the analyte.

Double competitive LFIA includes an additional parameter that determines the color intensity of the analytical zone and assay sensitivity. This parameter is the concentration of hapten-protein conjugate, which was mixed with the sample. Concurrently, the dependence of color intensity in the analytical zone and assay LOD on the hapten-protein concentration has a maximum. We used three CAP-BSA concentrations: 200, 400, and 800 ng/mL. For conjugate of GNP with antibodies in the amount of 10 µg/mL, the maximal color in the absence of the analyte was reached at CAP-BSA concentration of 400 ng/mL, with color intensity equal to 14.3 RU. A similar color intensity (15.5 RU) was achieved in the standard scheme when using conjugate of GNP with antibodies in the amount of 5 µg/mL during synthesis (Figure 4). These parameters were chosen to compare the two LFIA schemes.

**Figure 5:**
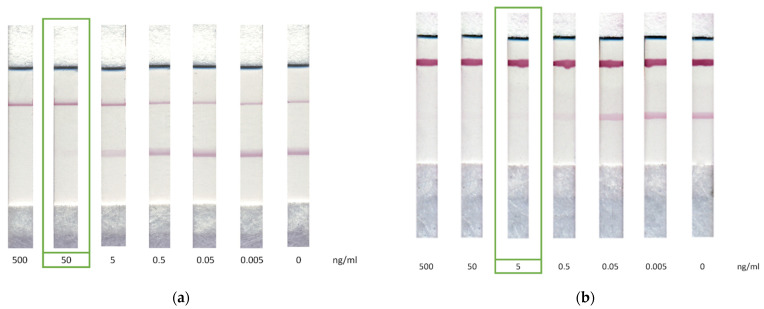
LFIA of CAP by (**a**) standard competitive scheme and (**b**) double competitive scheme. The appearance of test strips after the assays.

The LFIA for CAP solutions with concentrations from 0.005 to 500 ng/mL was carried out under the selected conditions. According to the obtained results, visual LOD, corresponding to the disappearance of coloration in the analytical zone, was 50 ng/mL (Figure 5a). The double competitive LFIA demonstrated visual LOD of 5 ng/mL (Figure 5b). Instrumental LOD, calculated according to the 3σ criterion, was observed at a concentration of 400 pg/mL and dynamic range from 0.28 to 10.88 ng/mL (R^2^ = 0.95874) and at a concentration of 20 pg/mL and dynamic range from 0.03 to 0.23 ng/mL (R^2^ = 0.99493) in the standard and proposed scheme, respectively (Figure 3b).

### 3.3. Assessment of the Developed Double Competitive LFIA in Honey Samples

**Figure 6:**
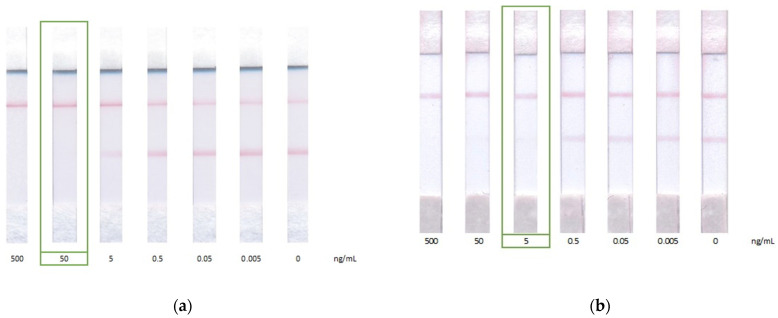
LFIA of CAP in honey by (**a**) standard competitive scheme and (**b**) double competitive scheme. The appearance of test strips after the assays.

**Figure 7:**
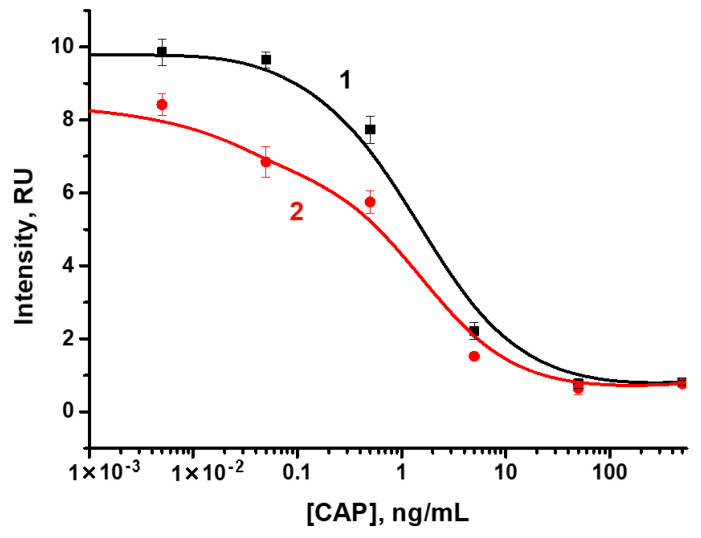
Color intensity in the analytical zones of test strips (from Figure 6) for the determination of CAP: (1) in the standard scheme of competitive LFIA; (2) in the proposed scheme of double competitive LFIA.

The developed double competitive LFIA was applied to test spiked honey samples, which were five-fold diluted with PBST. The dilution approach for sample preparation was used due to its rapidity, simplicity, and effectiveness, allowing reducing matrix interference. According to the obtained data (Figure 6), visual LOD, corresponding to the disappearance of coloration in the analytical zone, was 50 ng/mL in the standard scheme and 5 ng/mL in the proposed scheme. Instrumental LOD for CAP assay in honey was 300 pg/mL (R^2^ = 0.99926) or 1.2 µg/kg in conversion per sample weight of honey in the standard scheme, and 20 pg/mL (R^2^ = 0.96853) or 80 ng/kg in conversion per sample weight of honey in the proposed scheme, which meets the requirement of chloramphenicol MRL in honey of 0.3 mg/kg (Figure 7) [31].

## 4. Conclusions

A new competitive LFIA scheme has been proposed that provides highly sensitive detection of small molecules without the use of additional steps. Numerical simulation using COPASI software demonstrated that the given scheme is more sensitive compared to standard competitive LFIA. The proposed LFIA can be used for highly sensitive, rapid determination of low-molecular-weight compounds. The presented models and experimental confirmations also demonstrate that polyvalent interactions can be effective tools for improving the sensitivity of assays and can be used in the development of other types of analytical systems with increased sensitivity (for example, in ELISA).

## Data Availability

Data are contained within the article. Initial data of instrumental measurements are available on request from the corresponding author.

## Supplementary Materials

The following supporting information can be downloaded at: https://www.mdpi.com/article/10.3390/bios12050343/s1, Figure S1: Differential equations systems for describing the kinetics of affine interactions for standard competitive LFIA (A) and proposed alternative scheme of competitive immunochromatography (B); Figure S2: Parameters of the proposed model of double competitive LFIA.
