# Supplementary material for: Double Competitive Immunodetection of Small Analyte: Realization for Highly Sensitive Lateral Flow Immunoassay of Chloramphenicol
Source: Biosensors (Basel). 2022 May 17;12(5):343. doi: 10.3390/bios12050343 (PMC9138499; doi:10.3390/bios12050343)
Supplement: Supplementary file 1 [file biosensors-12-00343-s001.zip › biosensors-1723821-supplement.pdf]

Supplementary Information

Double Competitive Immunodetection of Small Analyte: Reali-Zation for Highly Sensitive Lateral Flow Immunoassay of Chlo-Ramphenicol

Dmitriy V. Sotnikov[1], Lyubov V. Barshevskaya[1], Anastasia V. Bartosh [1], Anatoly V. Zherdev[1] and Boris B. Dzantiev[1]*

[1] A.N. Bach Institute of Biochemistry, Research Center of Biotechnology of the Russian Academy of Sciences, Leninsky prospect 33, Moscow 119071, Russia

* Correspondence: dzantiev@inbi.ras.ru, Tel.: +7-495-954-31-42

$$\frac{d([P]\cdot V_{compartment})}{dt} = V_{compartment}\cdot(\text{"(reaction1).k2"}\cdot[AP] - \text{"(reaction1).k1"}\cdot[A]\cdot[P])$$

$$\frac{d([A]\cdot V_{compartment})}{dt} = V_{compartment}\cdot(\text{"(reaction1).k2"}\cdot[AP] - \text{"(reaction1).k1"}\cdot[P]\cdot[A])$$

$$\frac{d([AP]\cdot V_{compartment})}{dt} = V_{compartment}\cdot(\text{"(reaction1).k1"}\cdot[A]\cdot[P] - \text{"(reaction1).k2"}\cdot[AP])$$

$$\frac{d([R]\cdot V_{compartment})}{dt} = V_{compartment}\cdot((\text{"(reaction2).k2"}\cdot[PR] - \text{"(reaction2).k1"}\cdot[R]\cdot[P])\cdot h)$$

$$\frac{d([PR]\cdot V_{compartment})}{dt} = V_{compartment}\cdot((\text{"(reaction2).k1"}\cdot[R]\cdot[P] - \text{"(reaction2).k2"}\cdot[PR])\cdot h)$$

$$h = \begin{cases} \text{Time} < T, & 0 \\ \text{else}, & 1 \end{cases}$$

A

$$\frac{d([A]\cdot V_{compartment})}{dt} = V_{compartment}\cdot(\text{"(reaction\_1).k2"}\cdot[AP] - \text{"(reaction\_1).k1"}\cdot[A]\cdot[P])$$

$$\frac{d([AP]\cdot V_{compartment})}{dt} = V_{compartment}\cdot(\text{"(reaction\_1).k1"}\cdot[A]\cdot[P] - \text{"(reaction\_1).k2"}\cdot[AP])$$

$$\frac{d([P]\cdot V_{compartment})}{dt} = V_{compartment}\cdot(\text{"(reaction\_1).k2"}\cdot[AP] - \text{"(reaction\_1).k1"}\cdot[A]\cdot[P] + \text{"(reaction\_2).k2"}\cdot[PC] - \text{"(reaction\_2).k1"}\cdot[P]\cdot[C] + \text{"(reaction\_3).k2"}\cdot[PCP] - \text{"(reaction\_3).k1"}\cdot[P]\cdot[PC])$$

$$\frac{d([C]\cdot V_{compartment})}{dt} = V_{compartment}\cdot(\text{"(reaction\_2).k2"}\cdot[PC] - \text{"(reaction\_2).k1"}\cdot[P]\cdot[C])$$

$$\frac{d([PC]\cdot V_{compartment})}{dt} = V_{compartment}\cdot(\text{"(reaction\_2).k1"}\cdot[P]\cdot[C] - \text{"(reaction\_2).k2"}\cdot[PC] - \text{"(reaction\_3).k1"}\cdot[P]\cdot[PC] + \text{"(reaction\_3).k2"}\cdot[PCP])$$

$$\frac{d([PCP]\cdot V_{compartment})}{dt} = V_{compartment}\cdot(\text{"(reaction\_3).k1"}\cdot[P]\cdot[PC] - \text{"(reaction\_3).k2"}\cdot[PCP])$$

$$\frac{d([AR]\cdot V_{compartment})}{dt} = V_{compartment}\cdot((\text{"(reaction\_4).k1"}\cdot[A]\cdot[R] - \text{"(reaction\_4).k2"}\cdot[AR])\cdot h)$$

$$\frac{d([R]\cdot V_{compartment})}{dt} = V_{compartment}\cdot((\text{"(reaction\_4).k2"}\cdot[AR] - \text{"(reaction\_4).k1"}\cdot[A]\cdot[R] + \text{"(reaction\_5).k2"}\cdot[CR] - \text{"(reaction\_5).k1"}\cdot[C]\cdot[R] + \text{"(reaction\_6).k2"}\cdot[PCR] - \text{"(reaction\_6).k1"}\cdot[PC]\cdot[R])\cdot h)$$

$$\frac{d([CR]\cdot V_{compartment})}{dt} = V_{compartment}\cdot((\text{"(reaction\_5).k1"}\cdot[C]\cdot[R] - \text{"(reaction\_5).k2"}\cdot[CR] + \text{"(reaction\_7).k2"}\cdot[PCR] - \text{"(reaction\_7).k1"}\cdot[P]\cdot[CR])\cdot h)$$

$$\frac{d([PCR]\cdot V_{compartment})}{dt} = V_{compartment}\cdot((\text{"(reaction\_6).k1"}\cdot[PC]\cdot[R] - \text{"(reaction\_6).k2"}\cdot[PCR] + \text{"(reaction\_7).k1"}\cdot[P]\cdot[CR] - \text{"(reaction\_7).k2"}\cdot[PCR])\cdot h)$$

$$h = \begin{cases} \text{Time} < T, & 0 \\ \text{else}, & 1 \end{cases}$$

B

Figure S1. Differential equations systems for describing the kinetics of affine interactions for standard competitive LFIA (A) and proposed alternative scheme of competitive immunochromatography (B).

Species

#	Name	Compartment	Type	Unit	Initial Concentration [Unit]	Concentration [Unit]	Rate [Unit/s]	Reactions	Initial Expression [Unit]
1	B	compartment	ode	mmol/ml	1e-08	nan	nan	3	
2	BA	compartment	ode	mmol/ml	0	nan	nan	4	
3	A	compartment	ode	mmol/ml	1e-07	nan	nan	4	
4	ABA	compartment	ode	mmol/ml	0	nan	nan	1	
5	BP	compartment	ode	mmol/ml	0	nan	nan	4	
6	P	compartment	ode	mmol/ml	1e-08	nan	nan	4	
7	PBP	compartment	ode	mmol/ml	0	nan	nan	1	
8	ABP	compartment	ode	mmol/ml	0	nan	nan	2	
9	BR	compartment	ode	mmol/ml	0	nan	nan	3	
10	R	compartment	ode	mmol/ml	1e-08	nan	nan	3	
11	ABR	compartment	ode	mmol/ml	0	nan	nan	2	
12	PBR	compartment	ode	mmol/ml	0	nan	nan	2	

Reactions

#	Name	Reaction	Rate Law	Flux [mmol/s]	Noise Expression
1	reaction1	B + A = BA	Mass action (reversible)	nan	
2	reaction2	BA + A = ABA	Mass action (reversible)	nan	
3	reaction3	B + P = BP	Mass action (reversible)	nan	
4	reaction4	BP + P = PBP	Mass action (reversible)	nan	
5	reaction5	BA + P = ABP	Mass action (reversible)	nan	
6	reaction6	BP + A = ABP	Mass action (reversible)	nan	
7	reaction7	B + R = BR	Mass action (reversible)	nan	
8	reaction8	BR + A = ABR	Mass action (reversible)	nan	
9	reaction9	BR + P = PBR	Mass action (reversible)	nan	
10	reaction10	BA + R = ABR	Mass action (reversible)	nan	
11	reaction11	BP + R = PBR	Mass action (reversible)	nan	
	New Reaction				

Expression [Unit] or [Unit/s]
(reaction1).k2*[BA]-(reaction1).k1*[A]*[B]+((reaction3).k2*[BP]-(reaction3).k1*[B]*[P])*Values[h1]
(reaction1).k1*[B]*[A]+(reaction2).k2*[ABA]-(reaction1).k2*[BA]-(reaction2).k1*[BA]*[A]+((reaction5).k2*[ABP]-(reaction5).k1*[BA]*[P])*Values[h1]
(reaction1).k2*[BA]+(reaction2).k2*[ABA]-(reaction1).k1*[B]*[A]-(reaction2).k1*[A]*[BA]+((reaction6).k2*[ABP]-(reaction6).k1*[A]*[BP])*Values[h1]
(reaction2).k1*[A]*[BA]-(reaction2).k2*[ABA]
((reaction3).k1*[B]*[P]+(reaction4).k2*[PBP]+(reaction5).k2*[ABP]-(reaction3).k2*[BP]-(reaction4).k1*[BP]*[P]-(reaction6).k1*[A]*[BP])*Values[h1]
((reaction3).k2*[BP]+(reaction4).k2*[PBP]+(reaction5).k2*[ABP]-(reaction3).k1*[B]*[P]-(reaction4).k1*[BP]*[P]-(reaction5).k1*[BA]*[P])*Values[h1]
((reaction4).k1*[P]*[BP]-(reaction4).k2*[PBP])*Values[h1]
((reaction5).k1*[P]*[BA]+(reaction6).k1*[A]*[BP]-(reaction5).k2*[ABP]-(reaction6).k2*[ABP])*Values[h1]
((reaction7).k1*[B]*[R]+(reaction8).k2*[ABR]+(reaction9).k2*[PBR]-(reaction7).k2*[BR]-(reaction8).k1*[BR]*[A]-(reaction9).k1*[P]*[BR])*Values[h2]
((reaction7).k2*[BR]+(reaction10).k2*[ABR]+(reaction11).k2*[PBR]-(reaction7).k1*[B]*[R]-(reaction10).k1*[R]*[BA]-(reaction11).k1*[R]*[BP])*Values[h2]
((reaction8).k1*[BR]*[A]+(reaction10).k1*[R]*[BA]-(reaction8).k2*[ABR]-(reaction10).k2*[ABR])*Values[h2]
((reaction9).k1*[BR]*[P]+(reaction11).k1*[BP]*[R]-(reaction9).k2*[PBR]-(reaction11).k2*[PBR])*Values[h2]

Figure S2. Parameters of the proposed model of double competitive LFIA.
